# Inconclusive Diagnosis after Newborn Screening for Cystic Fibrosis

**DOI:** 10.3390/ijns6010019

**Published:** 2020-03-12

**Authors:** Anne Munck

**Affiliations:** Hopital Necker Enfants-Malades, AP-HP, CF centre, Université Paris Descartes, 75015 Paris, France; anne.munck1@gmail.com; Tel.: +33-60-9372-870

**Keywords:** cystic fibrosis, CF transmembrane conductance regulator-related metabolic syndrome, CF screen positive, inconclusive diagnosis, newborn screening

## Abstract

An unintended consequence of newborn screening for cystic fibrosis (CF) is the identification of infants with a positive screening test but an inconclusive diagnostic testing. These infants are designated as CF transmembrane conductance regulator-related metabolic syndrome (CRMS) in the US and CF screen-positive, inconclusive diagnosis (CFSPID) in Europe. Recently, experts agreed on a unified international definition of CRMS/CFSPID which will improve our knowledge on the epidemiology and outcomes of these infants and optimize comparisons between cohorts. Many of these children will remain free of symptoms, but a number may develop clinical features suggestive of CFTR-related disorder (CFTR-RD) or CF later in life. Clinicians should to be prepared to identify these infants and communicate with parents about this challenging and stressful situation for both healthcare professionals and families. In this review, we present the recent publications on infants designated as CRMS/CFSPID, including the definition, the incidence across Europe, the assessment of the CFTR protein function, the outcomes with the rates of conversion to a final diagnosis of CF and their management.

## 1. Introduction

Newborn screening (NBS) for cystic fibrosis (CF), when combined with very early multidisciplinary care at CF centers (CFC), is acknowledged as the optimal approach to CF diagnosis, as it maximizes the long-term prognosis and survival of these children [1,2,3]. However, beyond the goal of NBS and irrespective of the screening protocol used, there is the detection of infants with a positive NBS test and an inconclusive designation [4]. The terminology used for these infants is CF transmembrane conductance regulator-related metabolic syndrome (CRMS) in the US [5] and CF screen-positive, inconclusive diagnosis (CFSPID) in Europe [6]. Many of these children will remain asymptomatic, but later in life, a number of them may develop symptoms suggestive or CFTR-related disorder (CFTR-RD) or CF [7]. The approach to these infants is evolving as clinical experience grows; nevertheless, uncertainty remains challenging for families and caregivers 

## 2. Inconclusive Diagnosis after Newborn Screening

### 2.1. Definition of CRMS/CFSPID

**Figure 1:**
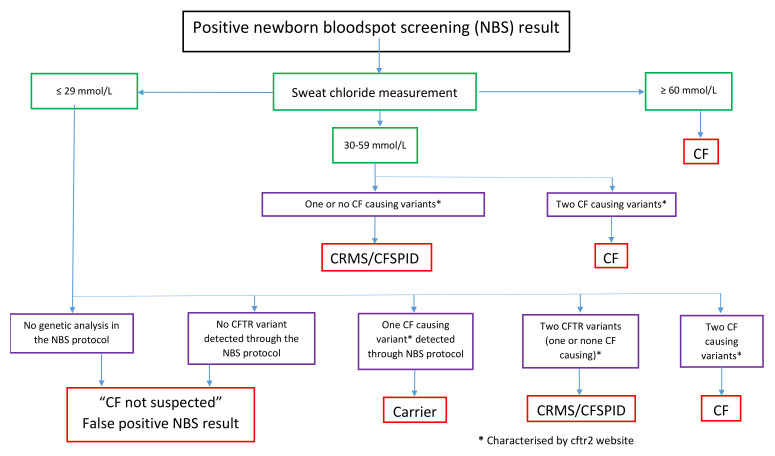
An algorithm for the designation of infants, following the positive newborn screening (NBS) result [9]. CF: Cystic fibrosis, CFTR: CF transmembrane conductance regulator (gene), CFMS: CFTR-related metabolic syndrome, CFSPID: CF screen-positive, inconclusive diagnosis.

For infants with a positive NBS test but an inconclusive diagnosis, a definition has been created using the terminology CRMS in the US since 2009 [5] that is included in the International Statistical Classification of Diseases and Related Health Problems, Ninth Revision medical code (277.9), which is mandatory in the US for healthcare delivery. Recently, in Europe, a Delphi process conducted by the European CF Society (ECFS) Neonatal Screening Working Group (NN WG) identified the need for a designation, and the terminology CFSPID was introduced in 2015 [6]. The differences between these two definitions were minor. To optimize comparisons between cohorts and thus improve our knowledge on the epidemiology and outcomes, experts from around the world gathered at a Diagnosis Consensus Conference held in the US, in 2015, and agreed on a unified definition (Table 1) of CRMS/CFSPID [8], with a recently published algorithm for this definition in Figure 1 [9]. This definition incorporates the knowledge on *CFTR* variants characteristics as “CF causing”, “non-CF causing”, “varying clinical consequences” or “unknown significance” [10] in the CFTR2 database, which is regularly updated and searchable on the website https://cftr2.org. However, an international survey conducted in 2018 by ECFS NN WG, with support of the Cystic Fibrosis Foundation (CFF) NBS Quality Improvement Group, showed significant confusion in regard to the correct designation of inconclusive diagnosis in six scenarios of infants screening positive. In one-third to half of the respondents, who were either CF doctors or pediatric pulmonologists [9], the diagnosis option was incorrect, thus identifying the need for improved education and communication. 

### 2.2. Incidence of CRMS/CFSPID across Europe

Within the two past decades, there has been a huge increase in NBS programs for CF worldwide, including in Europe. A recent European survey [4] reported data from 16 out of the 17 national NBS protocols with centralized data collection. Since then, national programs have been developed in Portugal (2015), Germany (2016), Denmark (2016), Macedonia (2018) and Belgium (2020); and Spain and Italy have regional programs that provide extensive coverage of the population. Strategies of NBS protocols and structure of programs vary widely, like the proportion of cases designated CRMS/CFSPID, reflecting the different approaches. This survey collected data during the year 2014, when the definition of CFSPID was not yet available, and therefore the recognition of infants may possibly be underestimated. The ratio of infants with CF compared to CFSPID ranged from 1.2:1 (Poland) to 32:1 (Ireland), and protocols, including larger panels of DNA mutations, were more likely to identify those infants. Minimizing the number of cases with CRMS/CFSPID remains an important consideration in NBS programs, as referring and following these infants create a burden for their families and healthcare professionals, and the benefits remain unclear.

### 2.3. Assessment of CFTR Protein Function

In cases where repeated sweat tests’ levels remain within the intermediate range, functional analyses measuring CFTR activity may help clarify the diagnosis. The assessment of the level of CFTR function is based on in vivo pharmacological studies, such as nasal potential difference (NPD) [11] or ex vivo intestinal current measurement (ICM) performed on rectal biopsies perfused in Ussing chambers [12], and on some occasion in combination with intestinal organoids analysis [13]. These evaluations of the CFTR function are performed exclusively in highly specialized CF centers and are not currently used in clinical practice. A diagnosis of CF can be ruled out when these functional analyses are within the normal range. The level of CFTR function further defines the likelihood of developing CF, as there is a continuum of CFTR dysfunction, and the paradigm of CF can be defined in terms of risk, depending on the severity of the dysfunction. 

## 3. Monitoring Infants Designated CRMS/CFSPID

### 3.1. Outcomes and Conversion to a Final Diagnosis of CF in Infants Designated CRMS/CFSPID

Infants designated CRMS/CFSPID may later develop a diagnosis of CFTR-RD, and a number may have symptoms suggestive of CF and convert to a final diagnosis of CF (a less classical form in most cases). The range of conversion to CF varied widely in retrospective or registry database studies [14,15,16,17,18], from 6% [14] to 48% [15] (Table 2). Neither the definition of cases with an inconclusive diagnosis nor the diagnosis criteria for conversion to CF was consistent among these studies, as well as the duration of follow-up, thus providing an explanation in this wide range of conversion to CF. Reclassification to CF should be based either on subsequent positive sweat test and/or two *CFTR* variants as CF causing *in trans* according to new knowledge acquired in CFTR2. Conversion to CF is also more likely related to individual CFTR variants [19,20,21] and to infants with an initial intermediate sweat chloride (SC) value compared to normal SC [16]. In two recent prospective studies, the conversion rate varied from 11% [19] to 44% [21]. The first prospective study was set up by Ooi et al. [19] in eight CF centers in Canada and Italy. Eighty-two positive NBS infants with an inconclusive diagnosis of CF, born 2007–2013, were matched 1:1 with a cohort diagnosed with CF through NBS (*n* = 80) and were evaluated at a median age of 2.2 years. Those with a CRMS/CFSPID designation at baseline had significantly lower median IRT (77 µg/L vs. 144 µg/L, *p* < 0.0001) and SC values (27.3 mmol/L vs. 83.2 mmol/L, *p* < 0.0001) compared to those diagnosed with CF. During follow-up, compared to those with CF, they all had sustained exocrine pancreatic sufficiency and less respiratory symptoms as well as identification of *Pseudomonas aeruginosa* (12% vs. 31%) and *Staphylococcus aureus* (40% vs. 70%). Among the 82 cases with a CRMS/CFSPID diagnosis, nine (11%) children converted to a delayed CF diagnosis based on positive SC value (*n* = 2), with the identification of two CF-causing mutations *in trans* in the CFTR2 database at the time of data analysis (*n* = 4) or both in three cases. Serial repeated sweat testing showed a mean age of 21.3 months at the time of conversion in those diagnosed with CF with a positive SC value. Those who converted to CF had higher initial SC values, no clinical or anthropometric differences and a trend toward more *Pseudomonas aeruginosa* identification compared to those who did not convert to CF. Authors shed light on the limited duration of follow-up with a caution in interpretation, as manifestations suggestive of CF may not develop until adolescence or adulthood. The same team [22] analyzed a larger cohort with CRMS/CFSPID and found a difference in initial NBS IRT median values in those with delayed CF (*n* = 14) compared to those who remained CRMS/CFSPID (*n* = 83), respectively with a median [Q1-Q3] of 108.9 (72.3–126.8) vs. 73.7 (60.0–96.0); *p* = 0.02, suggesting IRT initial value and trajectory over time as a potential tool to stratify young infants into high-risk or low-risk groups of developing CF. Munck et al. [21] reported a prospective multicenter study in France. Sixty-three positive NBS infants with an inconclusive diagnosis of CF, born 2002–2009, were matched 1:1 with a cohort diagnosed CF through NBS (*n* = 63) and evaluated at a mean age of 7.4 years. Those with a CRMS/CFSPID designation at baseline had a significantly lower median IRT (97 µg/L vs. 166 µg/L, *p* < 0.0001) and SC values (40 mmol/L vs. 110 mmol/L, *p* < 0.0001) compared to those diagnosed CF. During follow-up, compared to those with CF, they all had sustained exocrine pancreatic sufficiency, less respiratory symptoms and identification of *Pseudomonas aeruginosa* (24% vs. 82%) and *Staphylococcus aureus* (68% vs. 90%). Among the 63 cases with a CRMS/CFSPID diagnosis, 28 (44%) children converted to a delayed CF diagnosis based on a positive SC value (*n* = 8), with the identification of two CF-causing mutations *in trans* in the CFTR2 database at the time of data analysis (*n* = 12) or both in eight cases. All but six presented during follow-up respiratory symptoms suggestive of CF (productive cough, pathogens, antibiotic courses), although not specific to CF. Those who converted to CF had similar initial SC values, no clinical, anthropometric, respiratory pathogens, radiological or spirometry differences at final assessment compared to those who did not convert to CF. Infants recruited in these two studies reflected children currently diagnosed as CRMS/CFSPID, and the conversion to CF was defined by the above strict criteria. Explanations for the discrepancy in CF conversion rates among studies may be a time lag in infants’ birth dates with differences in updated CFTR2 knowledge at the time of data analysis, as well as variations in the duration of follow-up.

### 3.2. Management of Infants with CRMS/CFSPID Designation

Whether screening newborns for CRMS/CFSPID is of clinical benefit has yet to be established, as most of these infants seem unlikely to develop any phenotype. Those designated CRMS/CFSPID have no clinical feature suggestive of CF at initial evaluation. It is important to provide accurate information to parents who feel psychologically distressed with the delivery of an initial positive NBS result and an inconclusive designation [23]. For an appropriate follow-up, most CF physicians agree that a balance is needed to avoid both overmedicalization and undertreatment, which can be a missed opportunity to prevent manifestations later in life. Nevertheless, there is no evidence that early proactive treatment leads to better long-term outcomes. Considering the US guidelines [5] published in 2009 and European recommendations [21,24] published in 2009 and updated in 2015 on early management, they are in agreement on care issues and on regular follow-up by a physician with an interest in CF encouraging clinical assessment rather than unnecessary explorations and including regular sweat testing. Clear information should be provided to the family and the primary care physician over time. Ooi et al. [19] considered his study as an interim one according to the short duration of monitoring and that CF-like manifestations may not develop until adolescence or adulthood. Data of Munck et al. [21], with a longer monitoring period and a more comprehensive respiratory status assessment, support a less intensive approach in the management of these infants compared to those with CF. They consider a possible discharge from the CF center after six years of age if the child has not converted to CF with the primary care physician remaining vigilant, especially for unexplained chronic lung disease. The best practice for monitoring these children is still an unanswered issue. Both prospective studies shed light on the need of further long-term prospective studies. In parallel, the ECFS NN WG is now working on a consensual document for monitoring these individuals from initial assessment to six years of age, with a diagnostic testing section, a care management section, including respiratory phenotype, and a review of evidence from a year-six assessment, with shared a decision on future care plans with the family.

## 4. CRMS/CFSPID Registry Database

Analysis of the 2010–2012 CFF Patient Registry database by Ren et al. [15] showed a high rate of misclassification of NBS-positive infants. On one hand, 11% of infants with CRMS had to be reclassified as CF after expansion of the number of CF-causing mutations and/or subsequent positive SC; and on the other hand, 41% of infants with CRMS were assigned as CF, despite not fulfilling the criteria. Now with the unified definition for infants designated CRMS/CFSPID, we can speculate that registry databases monitoring long-term outcomes will provide an accurate assessment of the risk of moving through adolescence or adulthood to CFTR-RD and CF diagnosis and will contribute to better define the modalities of monitoring. The ECFS NN WG is now working with the ECFS Registry team to prepare a European survey for infants with a designation of CRMS/CFSPID, aimed at recording the current situation of existing national or regional registries or databases, or plans and timelines to develop them.

## Figures and Tables

**Table 1:** Definitions for CF transmembrane conductance regulator-related metabolic syndrome (CRMS) and CF screen-positive, inconclusive diagnosis (CFSPID) and the harmonized definition CRMS/CFSPID.

	Positive NBS	And	Or
CRMS [5] US	Asymptomatic infants with hypertrypsinemia at birth	Persistently intermediate sweat chloride levels ^1^ and fewer than 2 CF-causing CFTR mutations	Sweat chloride concentration <30 mmol/L and 2 CFTR mutations with 0 or 1 known to be CF-causing
CFSPID [6] Europe	Asymptomatic infants with hypertrypsinemia at birth	0 or 1 CFTR mutation, plus intermediate sweat chloride (30–59 mmol/L)	2 CFTR mutations, at least 1 of which has unclear phenotypic consequences, plus a normal sweat chloride (<30 mmol/L)
CRMS/CFSPID [8]	Infants with positive newborn screening test	Sweat chloride <30 mmol/L and 2 CFTR mutations with 0 or 1 CF-causing CFTR mutation	Sweat chloride 30–59 mmol/L and 0 or 1 CF-causing CFTR mutation

^1^ Sweat chloride levels: 30–59 mmol/L if age < 6 months or 40–59 mmol/L if age ≥6 months.

**Table 2:** Summary of recent studies of CRMS/CFSPID.

	Kharrazi et al. [14]	Groves et al. [15]	Ren et al. [16]	Levy et al. [17]	Terlizzi et al. [18]	Ooi et al. [19]	Munck et al. [21]
Study design	Retrospective	Retrospective case control	CFF registry	Cross sectional	Retrospective	Prospective case control	Prospective case control
Country	USA California	Australia	US	US Wisconsin	Italy Tuscany	Canada, Italy	France
Birth period	2007–2012	1996–2010	2010–2012	1994–2012	2011–2016	2007–2013	2002–2009
Follow up duration (y)	Mean 4.5	10	1	8	Median 0.6	Median 2.2	Mean 7.4
Number CF	345	225	1540	300	32	80	63
Number CRMS/CFSPID	533	29 ^2^	309	57	50	82	63 ^2^
CF:CRMS/CFSPID	0.65:1	7.8:1	5:1	5.2:1	0.64:1	1.8:1 ^6^	6.3:1 ^6^
Conversion to CF, N (%)	20 (5.8)	14/29 (48) matched to CF	NA ^4^	NA ^4^	5 (10)	9 (11)	28(44)
Increased SCC ≥60 mmol/L	17	2 ^3^			5	2	8
2 CF causing mutations	0	0			0	4	12
Both criteria	0	0			0	3	8
Other criteria	3	12			0	0	0
Age at conversion (y)	Mean 2.5 ± 1.4				Median 2 (0.2–4)	Mean 1.8 ± 1.2	Unk ^1^
Pseudomonas aeruginosa, N (%)	Unk ^1^	78.6	10.7	39	25 ^5^	12	24
Pancreatic insufficiency, N (%)	3/15 (15)	4/29 (14)	14/309 (4.5)	0	0	0	0
F508del/R117H, N (%)	Unk ^1^	4/14 (29)	80/309 (26)	37/57 (63)	0	16/82 (19.5)	27/63 (43)

^1^ Unk: unknown; ^2^ definition slightly different from CRMS/CFSPID; ^3^ only 8/14 had a repeated sweat test; ^4^ NA: non-applicable; ^5^ only 8/50 had swab culture. CF: ^6^ CF: CRMS/CFSPID ratio from the algorithm. Cystic fibrosis, CFTR: CF transmembrane conductance regulator (gene), CFMS: CFTR-related metabolic syndrome, CFSPID: CF screen-positive, inconclusive diagnosis, SCC: sweat chloride concentration
